# Supplementary figures and images for: Yersinia remodels epigenetic histone modifications in human macrophages
Source: PLoS Pathog. 2021 Nov 18;17(11):e1010074. doi: 10.1371/journal.ppat.1010074 (PMC8639070; doi:10.1371/journal.ppat.1010074)

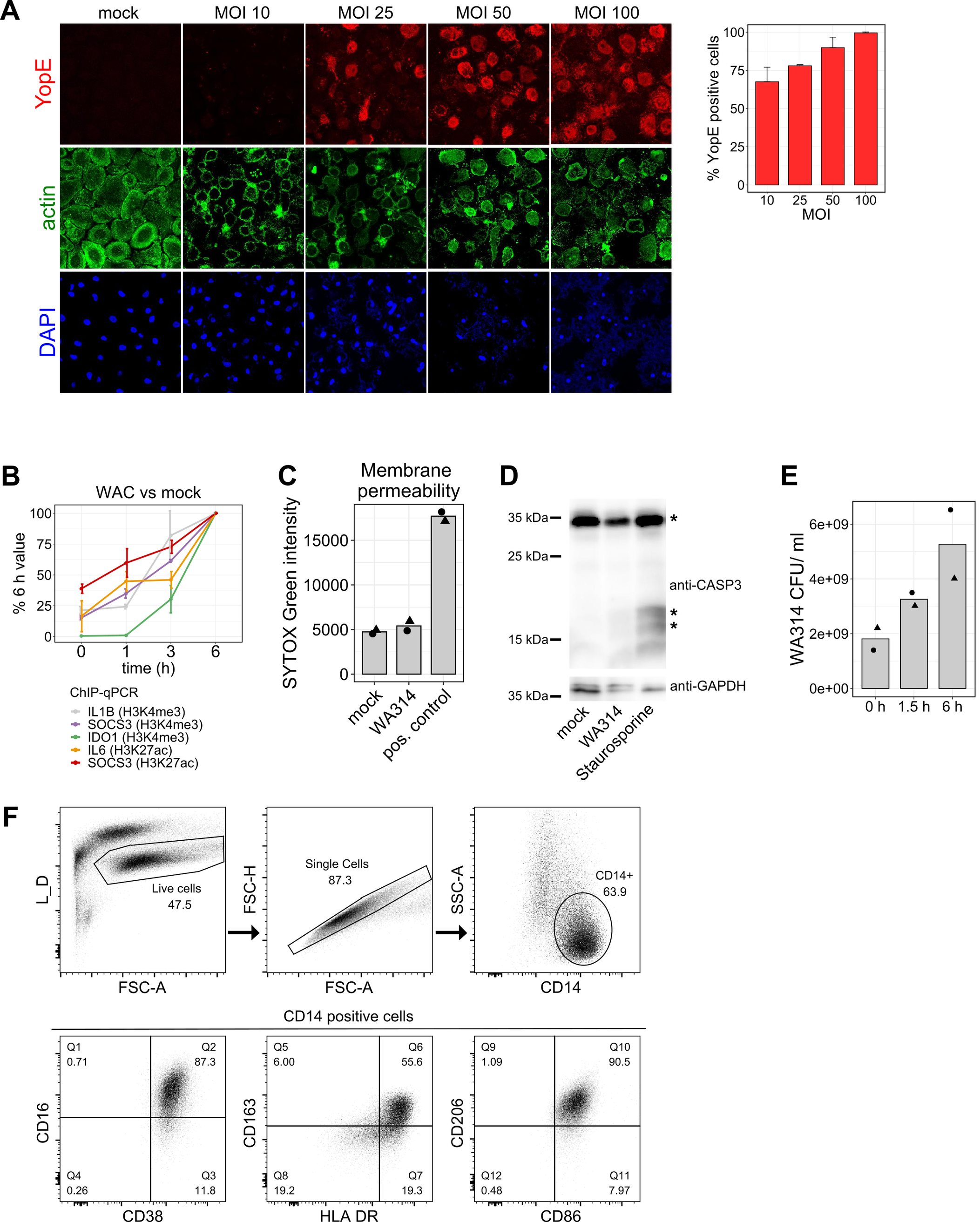

Supplement: S1 Fig — A, Left: Immunofluorescence staining of primary human macrophages mock infected or infected with WA314-YopE-ALFA for 6 h with MOI 10, 25, 50 or 100. Cells were stained with anti-ALFA nanobody-635 (red) to visualize YopE, Alexa568 phalloidin (green) to visualize actin and DAPI (blue) to visualize nucleus. Right: Quantification of YopE-ALFA positive cells. Bars show mean and error bars represent standard deviation when analyzing two different coverslips. B, Line plot showing time course H3K4me3 (IL1B, SOCS3, IDO1 genes) and H3K27ac (IL6, SOCS3 genes) ChIP-qPCR analysis of WAC infected primary human macrophages with MOI of 100. Lines represent means and error bars represent standard deviation from two independent macrophage donors/ biological replicates. C, Bar plot showing membrane permeability due to cell death in primary human macrophages mock infected, infected with WA314 (MOI of 100) or treated with positive (pos.) control (1% TRITON-X-100) for 6 h. Increased membrane permeability is expressed as higher SYTOX Green intensity, where the dye binds to DNA and emits fluorescence when plasma membrane integrity is compromised. Bars represent means of two biological replicates/ macrophage donors (dots with different shapes). D, Western blot analysis of primary human macrophages showing caspase-3 cleavage in mock or WA314 (MOI 100) infected cells or treated with positive control Staurosporine (5 μM) for 6 h. * indicates full length caspase-3 (35 kDa) and cleavage fragments (17 and 19 kDa). GAPDH was used as a loading control. Data are representative of four biological replicates/ different macrophage donors. E, Bar plot showing colony forming unit (CFU) analysis of Y. enterocolitica WA314 growth during infection of primary human macrophages for 0 h, 1.5 h or 6 h at 37°C. Bars represent means of two different biological replicates (dots with different shapes). F, Flow cytometry analysis of monocyte-derived primary human macrophages after 6 days of differentiation. Live cel [file ppat.1010074.s001.tif]

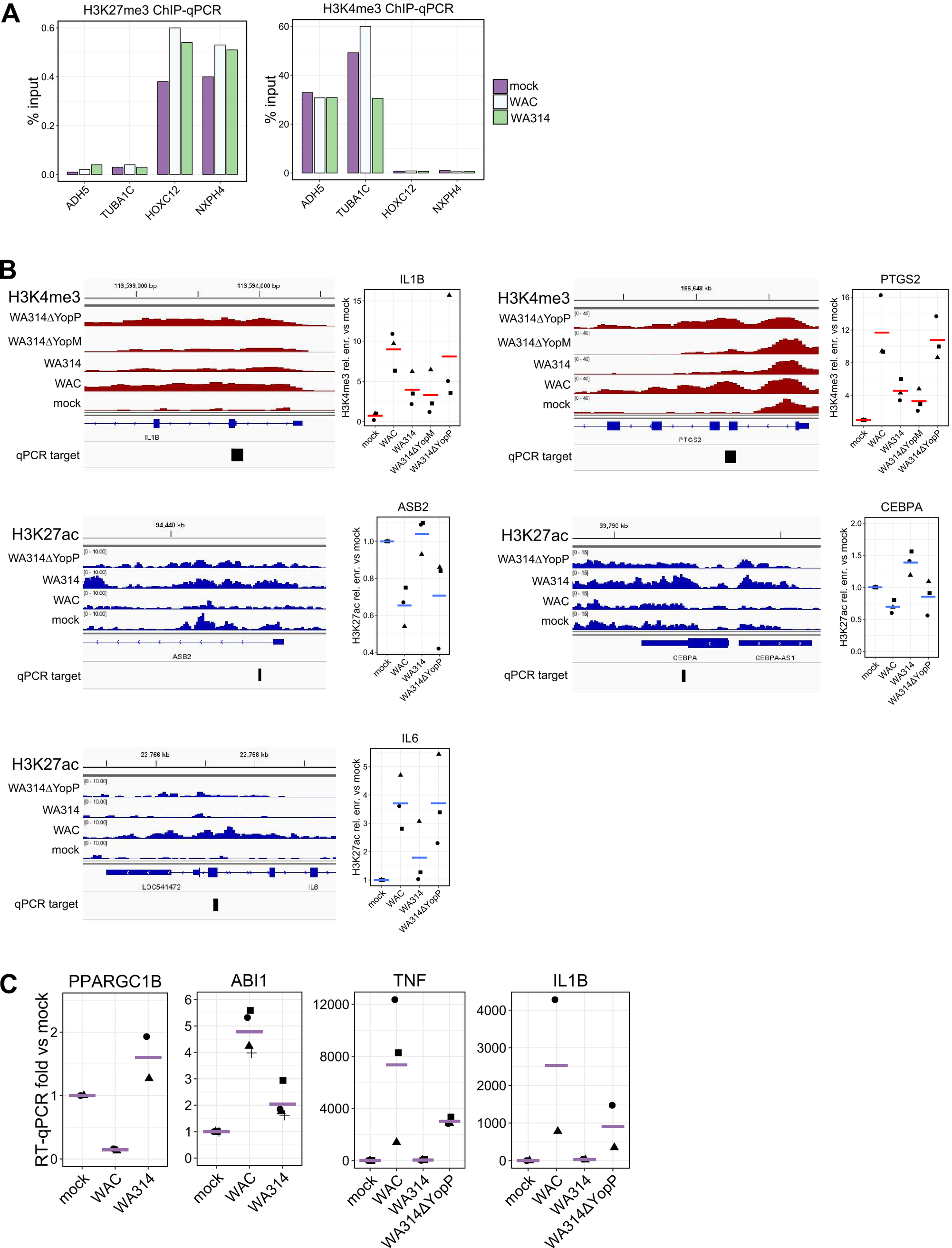

Supplement: S2 Fig — A, Bar plot showing H3K27me3 (left) and H4K4me3 (right) ChIP-qPCR from primary human macrophages mock infected or infected with WAC or WA314 for 6 h with MOI of 100. HOXC12 and NXPH4 are positive controls for H3K27me3 and ADH5 and TUBA1C are positive controls for H3K4me3. Data are representative of at least two experiments with different biological replicates/ macrophage donors. B, Peak tracks showing tag density of H3K4me3 (red) or H3K27ac (blue) ChIP-seq from primary human macrophages infected for 6 h with indicated strains (MOI of 100) (left). qPCR target refers to the site used for fragment amplification in ChIP-qPCR analysis shown as a dot plot (right). The ChIP-qPCR signal was expressed as relative (rel.) enrichment (enr.) vs mock. Lines represent means from at least two different biological replicates/ macrophage donors (dots with different shapes). C, Dot plots showing gene expression analysis of PPARGC1B, ABI1, TNF and IL1B genes with RT-qPCR from primary human macrophages infected for 6 h with indicated strains (MOI 100). The RT-qPCR signal was expressed as fold vs mock. Lines represent means from at least two different biological replicates/ macrophage donors (dots with different shapes). (TIF) [file ppat.1010074.s002.tif]

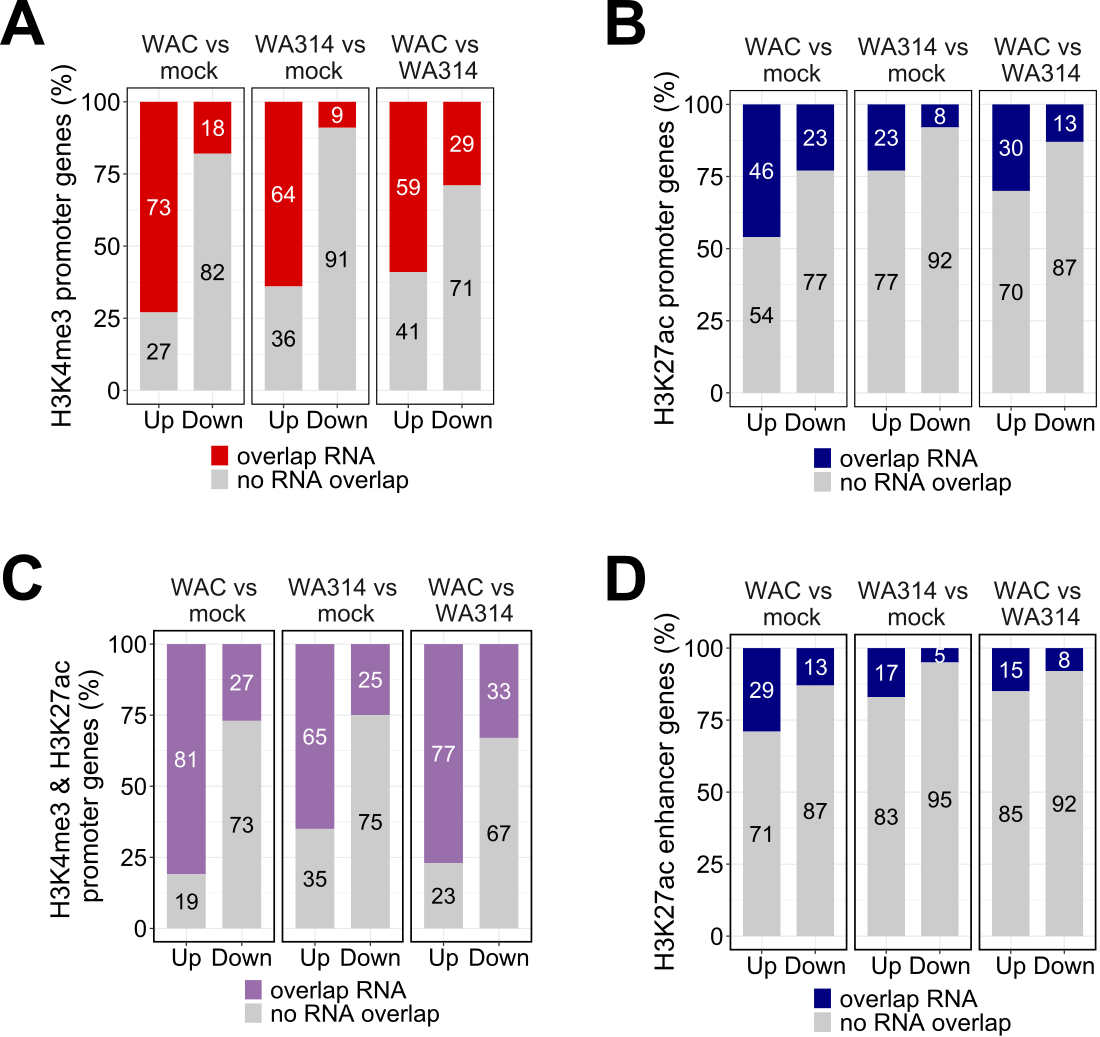

Supplement: S3 Fig — A-D, Bar plots showing fraction (%) of genes associated with H3K4me3 at promoter (A), H3K27ac at promoter (B), H3K4me3 and H3K27ac at promoter (C) and H3K27ac at enhancer (D) changes for comparisons between mock, WAC and WA314 showing significant (≥ 2-fold change, adjusted P-value ≤ 0.05) associated change in gene expression (overlap RNA). (TIF) [file ppat.1010074.s003.tif]

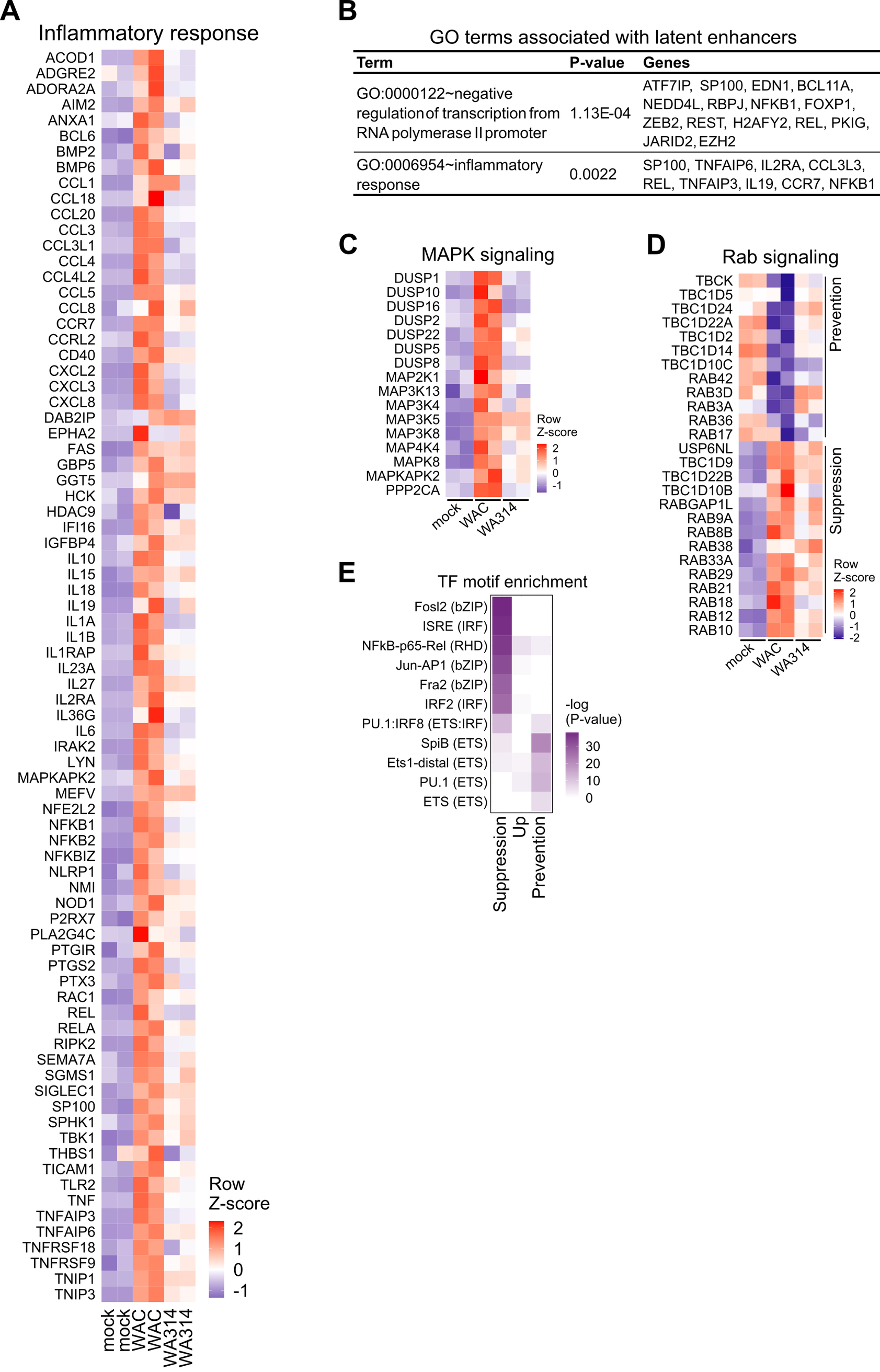

Supplement: S4 Fig — A, Heatmap of row-scaled (row Z-score) RNA-seq rlog gene counts for genes with RNA-seq and ChIP-seq promoter or enhancer overlaps belonging to “Inflammatory response” pathway as in Fig 3H. B, GO terms associated with latent enhancer genes from Fig 2F. C-D, Heatmaps of row-scaled (row Z-score) RNA-seq rlog gene counts for selected genes with RNA-seq and ChIP-seq promoter or enhancer overlaps belonging to phosphatases and kinases from MAPK signaling in Suppression profile (C) and Rab GTPase pathway in Prevention and Suppression profiles (D) from S16 Table. E, Heatmap showing transcription factor motif enrichment in promoter and enhancer regions of genes with RNA-seq and ChIP-seq overlaps from Fig 3G. Color indicates the level of log10 transformed P-value positively correlating with significance of enrichment. (TIF) [file ppat.1010074.s004.tif]

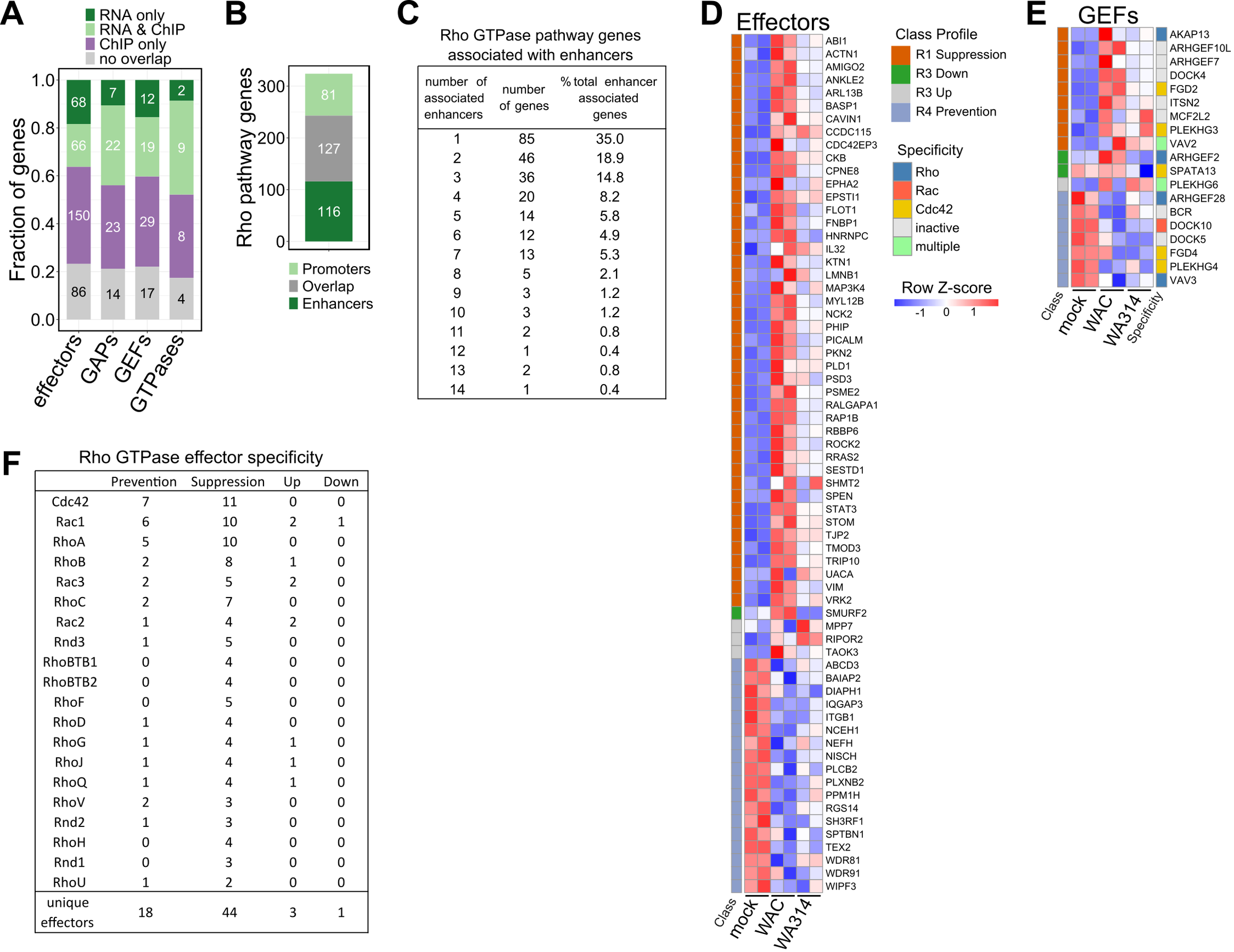

Supplement: S5 Fig — A, Bar plot showing fraction and number (in bars) of indicated Rho GTPase pathway genes with changes in expression only (RNA only), histone modification only (ChIP only), overlaps between RNA-seq and ChIP-seq (RNA & ChIP) and no overlap with neither ChIP-seq nor RNA-seq (no overlap). B, Bar plot showing number of Rho GTPase pathway genes with dynamic histone modifications at promoters, enhancers or both (overlap). C, Table showing number of Rho GTPase pathway genes with histone modification changes at enhancers and number of associated enhancers. “% total enhancer associated genes” indicates what fraction of all Rho GTPase pathway genes with changes at enhancers show a certain number of changed enhancers. D, E, Heatmaps of DEGs with associated histone modification changes at promoters or enhancers encoding Rho GTPase effectors (D) and GEFs (E). Associated classes are color coded on the left. The specificity of GEFs for Rho GTPases is color coded on the right (E). Gene rlog counts were row-scaled (row Z-score). F, Table showing specificity of Rho GTPase effectors associated with RNA-seq and ChIP-seq overlaps in (A) and Fig 4C. (TIF) [file ppat.1010074.s005.tif]

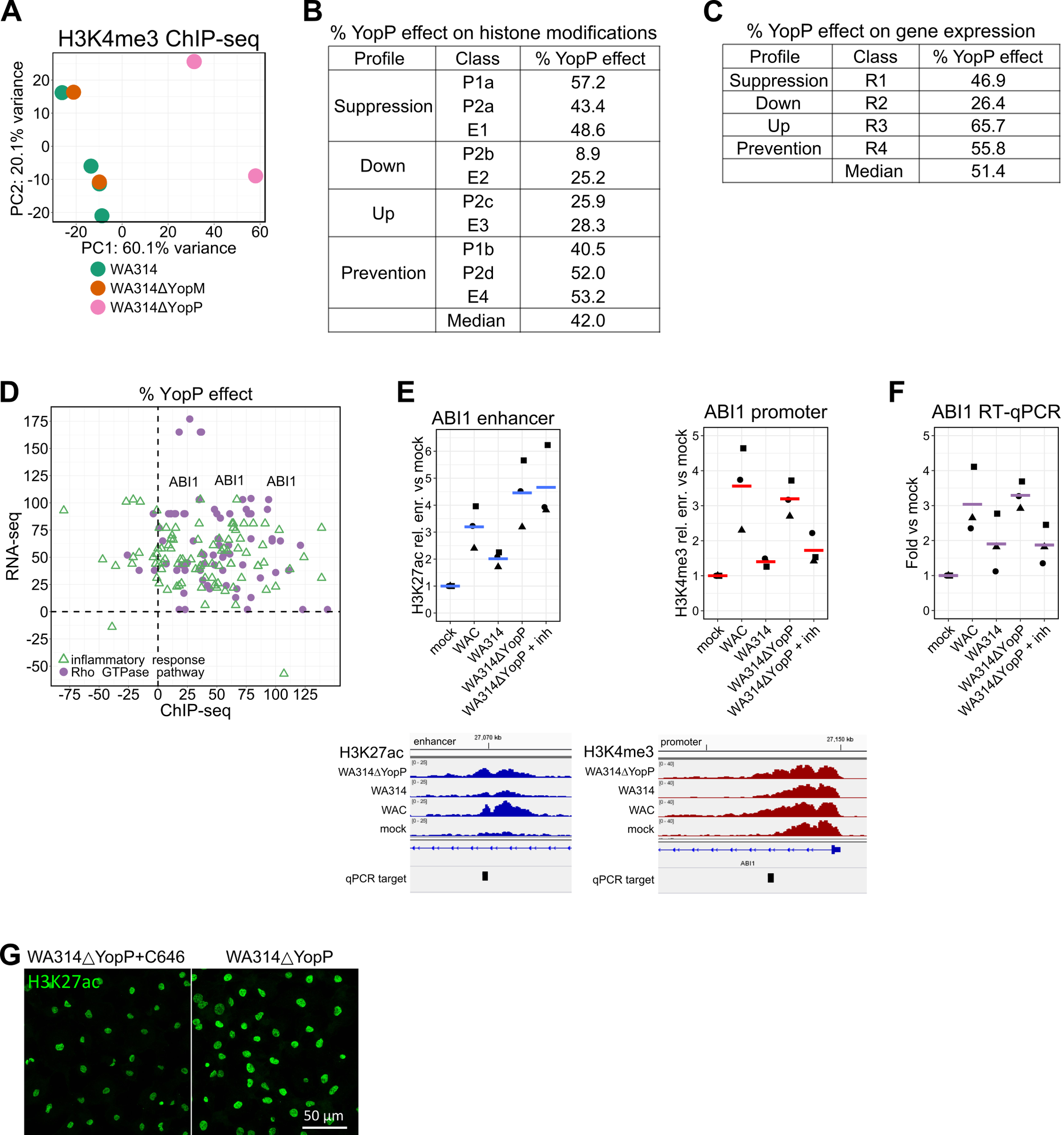

Supplement: S6 Fig — A, Principal component analysis of H3K4me3 tag counts in classes P1a-b of macrophages infected with the indicated strains for all biological replicates used in the analysis. B, Percentage YopP effect (median value) for H3K4me3 (P1a-b classes) and H3K27ac (P2a-d, E1-4 classes) when compared to WA314 vs mock (Up and Down profiles) or WA314 vs WAC (Suppression and Prevention profiles). C, Percentage YopP effect (median value) for DEGs associated with respective histone modification changes at promoters or enhancers. “Median”: median value when taking % YopP effect from all classes together. D, Scatter plot of % YopP effect for RNA-seq DEGs and ChIP-seq differential regions for genes with associated gene expression and histone modification change from Rho GTPase and Inflammatory response pathways from Fig 5C and 5D. Purple dots associated with ABI1 gene are indicated. E, Top: Dot plots showing ChIP-qPCR of H3K27ac at enhancer within ABI1 gene and H3K4me3 at ABI1 promoter from primary human macrophages infected for 6 h with indicated strains (MOI of 100). Lines represent means from three different biological replicates/ macrophage donors (dots with different shapes). Bottom: Peak tracks showing tag density of H3K27ac at enhancer (blue) and H3K4me3 at promoter (red) from ChIP-seq and site used for fragment amplification in ChIP-qPCR analysis (qPCR target) shown in dot plots. The ChIP-qPCR signal was expressed as relative (rel.) enrichment (enr.) vs mock. inh: MAPK inhibitors. F, Dot plots showing RT-qPCR gene expression analysis of ABI1 from primary human macrophages infected for 6 h with indicated strains (MOI of 100). The RT-qPCR signal was expressed as fold vs mock. Lines represent means from three different biological replicates/ macrophage donors (dots with different shapes). inh: MAPK inhibitors. G, Immunofluorescence staining of primary human macrophages infected with WA314ΔYopP+C646 (p300 inhibitor) or WA314ΔYopP with MOI of 100 for 6 h. Cells were stained with an [file ppat.1010074.s006.tif]
