# Supplementary material for: Yersinia remodels epigenetic histone modifications in human macrophages
Source: PLoS Pathog. 2021 Nov 18;17(11):e1010074. doi: 10.1371/journal.ppat.1010074 (PMC8639070; doi:10.1371/journal.ppat.1010074)
Supplement: Attachment — Submitted filename: Letter.docx [file ppat.1010074.s025.docx]

Dear Editor,

We thank you and the Reviewers for the time and comments in evaluating our manuscript. We have addressed all the Reviewers comments (see below) and we have added 17 new figures (mainly in supplementary material) including experiments for qPCR, immunofluorescence, cell death analysis, flow cytometry and bacterial growth. As suggested by the Reviewers we have changed the organization of the manuscript considerably. We hope that you will also find the revised manuscript much improved and can now accept it for publication in PloS Pathogens.

Sincerely,

Indra Bekere and Martin Aepfelbacher

**Answers to the Editor’s points**

**Please demonstrate the maturity and purity of the macrophages that you used.**

We have now included flow cytometry analysis in the S1F Fig analyzing expression of several known macrophage surface markers. We show that the macrophages in our culture are to 64 % CD14 positive and express known macrophage markers like CD163, CD38, CD86, HLA-DR,CD206 and CD16. These characteristics are typical for in vitro differentiated primary human macrophages. Please refer to the answer to the Reviewer #1: Methods, comment 2 (Part II) below for a more detailed answer to this point and a description of the according changes made to the manuscript.

**I agree with reviewer 2 regarding the problems associated with analyses being based on two biological replicates, please address with additional replicate(s).**

We have used 2-4 replicates for the experiments and demonstrate by clustering and PCA analysis (please refer to the answer to the Reviewer #2, comment 1 in Part II) that in all cases they showed good reproducibility. A minimum of 2 and more replicates are commonly used and are standard for ChIP-seq and RNA-seq experiments analyzing macrophage responses to stimulation. Further, the bioinformatic software employed is particularly well suited to work with 2-3 replicates. Therefore, we altogether argue that for the scope of this study the number of replicates used for RNA-seq and ChIP-seq is absolutely sufficient. Please refer to to the answer to the Reviewer #2, comment 1 (Part II) for a detailed argumentation of this issue and a description of the changes made to the manuscript, including a table detailing the number of replicates employed in the different experiments.

**Please include validation of detected histone modification marks and gene expression.**

Validation of histone marks from ChIP-seq and gene expression from RNA-seq is now included in S2 Fig, S6E, F Figs and Fig 5F. The qPCR data confirmed our ChIP-seq and RNA-seq analysis.

**Please address reviewer 3’s concern regarding evidence that histone modification is responsible for differential ABI1 gene expression and linked to actin cytoskeleton organization in Yersinia-infected macrophages.**

We have addressed this concern and toned down our statement that reorganization of actin cytoskeleton is histone modification-driven. We have also added additional ChIP-qPCR (S6E Fig) and RT-qPCR (S6F Fig) analysis of ABI1 gene overall showing that changes in histone modifications, gene expression, protein levels and redistribution of actin cytoskeleton and ABI1 are associated with MAPK pathway activation. Moreover, we also show that redistribution of ABI1 and actin is reversed by the treatment with p300 histone acetyltransferase inhibitor, which reduces H3K27ac (Fig 5J, S6G Fig).This experiment provides a more direct, albeit still not very specific, connection between the regulation of actin through histone modifications. Overall, these data well support the notion of histone modifications affecting ABI1 and actin cytoskeleton.

**Answers to the reviewer's comments and questions**

**Part I - Summary**

**Reviewer #1:** *In the manuscript entitled Epigenetic reprogramming of human macrophages by an intestinal pathogen, the authors provide a large amount of data on histone modifications induced by Yersinia enterocolitica during infection of human macrophages. The data presented here include genome wide ChIP-seq data on 4 different histone marks under 4 different conditions, as well as expression data for the 4 different conditions. In general, the data is of interest and constitutes a valuable resource, however the way in which the data is presented and the organization of the manuscript hinder the biological messages and take away from the significance of the results. As it stands, the paper is difficult to read, the figures are confusing, and the biological significance is lost and hard to follow. Therefore, major revisions in data presentation and contextual revisions are needed across text and methods.*

We thank the reviewer for his/ her valuable opinion and we have tried to address your comments to make the manuscript easier to read and grasp the main biological findings.

Our aim was to perform comprehensive analysis of gene expression and histone modifications to gain new insights in virulence mechanisms of *Yersinia enterocolitica.* For this we generated a large amount of ChIP-seq and RNA-seq data from different conditions. In our complex analysis we tried to extract and present the most interesting findings in an easily understandable way while integrating information from different datasets. Following your comment to improve the readability of the manuscript we have reorganized figures: i) grouped together promoter and enhancer analysis in Fig 2; ii) grouped together gene expression and pathway analysis in Fig 3 and iii) added several supplementary figures. To emphasize the biological meaning we have changed section titles in Results, added more pathway analysis (S4C, D Figs) and added a summary Figure 6. We think even though the investigated matter and results remain highly complex, the revised manuscript is now easier to interpret.

**Reviewer #2:** *The present study represents a global analysis of epigenetic histone modifications in primary human macrophages by the enteropathogenic pathogen Yersinia enterocolitica using genome-wide chromatin immunoprecipitation (ChIP) sequencing (CHIP-seq) which is compared with a parallel transcriptomic analysis (RNA-seq). The authors document that Y. enterocolitica infections are associated with major alterations of the chromatin state (H3K4me1, H3K4me3 and H3K27ac) of macrophage gene promoters and enhancers, and that the extent of the modification is manipulated by Y. enterocolitica pathogenicity factors encoded on the Yersinia virulence plasmid. In particular, YopJ/P is implicated in the reorganization of epigenetic histone modifications and associated gene reprogramming. Notably, many alterations appear to influence immune responses which are associated with Rho GTPase pathways.
The analyses of the data of the experiments in this study is very complex. To illustrate and interpret the results, the authors classified them in four logical profiles: (i) ‘Suppression’ and ‘Prevention’ for Yersinia-induced deposition or removal of histone marks and (ii) ‘Down’ and ‘Up’ in which the virulence plasmid-encoded pathogenicity factors down- or upregulate histone marks independently of other chromosomally encoded PAMP activities. This categorization is very helpful and indicates certain but also largely varying correlations of the histone modification and the actual gene expression pattern (30% Suppression to 2% Down profile).*

We thank the reviewer for his/ her positive comment and appreciate particularly that he/ she finds our categorization in profiles helpful. We are aware that we present a complex analysis and we have put a lot of effort in the revised manuscript to make it more readable. We think that varying degree of correlation between histone modifications and gene expression represents an interesting finding, indicating that a lot of “silent” modifications are induced during infection. We briefly discuss that these “silent” modifications might play a role in other functions than gene expression in the Discussion.

**Reviewer #3:** *This manuscript describes a comprehensive analysis of epigenetic histone modification changes in human macrophages infected with wild-type Yersinia enterocolitica or corresponding mutants lacking the plasmid encoding the type III secretion system or the effector YopP. The results provide new information to the field and are interesting. I have one comment about a weakness in data presentation and interpretation and several minor editorial suggestions.*

We thank the reviewer that he/ she finds the data in our manuscript new and interesting. We have responded to his/ her comment and changed the data presentation for ABI1 western blot analysis and manuscript text describing that “*that the histone modification-driven reprogramming of ABI1 gene expression in fact can alter actin cytoskeleton organization in Yersinia infected macrophages*”. We have also added additional data overall showing that H3K4me3 histone modification, gene expression and protein levels of ABI1 and accumulation of actin and ABI1 at cell cell contacts are regulated by MAPK pathway. Furthermore, we additionally show that inhibition of histone acetylation affects distribution of ABI1 and actin (Fig 5J, S6G Fig).

**Part II – Major Issues: Key Experiments Required for Acceptance**

**Reviewer #1: Methods:**
***1)*** *It would be helpful if more information was included on the infection phenotype of the macrophages that have been studied. Why was this MOI chosen? Did the authors use a gentamicin protection assay? If not, what is the bacterial growth over this period, which is likely multiple logs. Did the authors check for bacterial uptake – even though the T3SS is primed, macrophages may still internalize bacteria. What % at 6 h post-infection was internal vs. external? This information is important as the high MOI used for a 6h infection could be problematic. Furthermore, the authors mention that “at 6 h after infection, a time point at which a maximal effect on gene expression but no signs of apoptosis are detectable in the human macrophages”, but no data is shown on this point.*

We fully agree with the reviewer that the manuscript would profit from a more detailed description of the infection conditions, the logic why these conditions were chosen and a statement about the growth of the bacteria during macrophage infection.

i) The MOI of 100 was chosen because it resulted in 100 % of cells being positive for YopE, serving as a representative for Yop translocation (S1A Fig). We considered this to be advantageous for ensuring homogeneous transcriptional and epigenetic responses in the macrophage population.

ii) Analysis of the bacterial growth over 6 h showed that there was only a 3-fold increase in bacterial numbers (S1E Fig; described in Materials and Methods in CFU assay). We consider this minor increase unproblematic and therefore decided not to expose the primary macrophages to antibiotics avoiding potential side effects of these drugs.

iii) Data from our previous study (Nauth & Huschka et al., 2018 PLOS Pathogens) showed that macrophages still internalize bacteria even if the T3SS is primed.

iv) We found no signs of cell death after 6 h of infection (S1C, D Figs). We have analyzed membrane damage by measuring membrane permeability with SYTOX Green dye (S1C Fig) as a marker of necrosis and caspase-3 cleavage (S1D Fig) as a marker of apoptosis.

***2)*** *Please demonstrate the maturity and purity of the macrophages used in these studies. We agree that the method used from Linder et al (magnetic bead isolation by CD14) will collect monocytes, and then with serum and routine media changes to remove non-adherent cells the authors would generate macrophages. However, as stated in the methods that “Macrophages were used for infection 1-2 weeks after isolation” implies a large range of macrophage age, maturity, and responsiveness to stimulus. It would be important to show that macrophages used during this period are homogenous across time.*

We used standard purification and maturation protocols established in the laboratory for decades for obtaining pure human macrophages. Comparison of different monocyte isolation methods showed that CD14 positive selection of PBMCs, which was also employed in our study, yields highly pure and mature macrophages after differentiation (Nielsen et al., 2020 Immunology)*.* We have now included flow cytometry analysis in S1F Fig (described in Materials and Methods in Flow cytometry) showing that after isolation of PBMCs and differentiation into macrophages, 64 % of these macrophages are positive for the CD14 marker. This is in line with a recent study (Nielsen et al., 2020 Immunology) showing that CD14 positive isolation results in 52.6-85.5 % of cells expressing macrophage markers. We also showed that CD14 positive macrophages express macrophage markers CD163, CD38, CD86, HLA-DR,CD206 and CD16.

We apologize for not having been concise about the age of the macrophages used for the different experiments. In fact, most macrophages were 7-8 days old. Thus, all the ChIP-seq experiments and RNA-seq of samples from batch 1 (2 replicates of mock, WAC and WA314 infected macrophages described in Table S17) were performed with 7-8 days old macrophages. Only the additional RNA-seq experiments of samples from batch 2 (2 replicates of mock, WA314, WA314△YopM and WA314△YopP infected macrophages described in Table S17) were performed with 14 days old macrophages. This information is also now added in Materials and Methods section Cell culture. We previously tested whether gene expression profiles from 7 days vs. 14 days old macrophages are different and show that these profiles are strongly correlated (Figure below), indicating that employing 7 or 14 days old macrophages does not alter any results or conclusions of our experiments.

**Right: H**eatmap of RNA-seq sample clustering based on Euclidean distance where dark blue indicates smaller sample distances and larger similarity. **Left:** Principal component analysis of all RNA-seq sample replicates used for the analysis. All replicates used for the DEG analysis and clustering are shown. Normalized counts (rlog counts from DESeq2) of all genes used for DEG analysis were used (16859 genes). 1w or 2w indicates whether macrophages are 1 or 2 weeks old, respectively.

**Contextual changes:**
***3)*** *The following statement needs to be amended to include additional literature, and should be noted as it reads the authors are implying that because Yersinia replication extracellularly in draining lymph nodes (a hallmark of disease) the organism may suppress innate immune functions (etc…). This is not an accurate description, as part of the pathogenesis cycle does include intracellular niche depending upon the route of colonization of the host for all Yersinia spp. Perhaps it is more prudent to subjectively mention this with appropriate citations when applicable (St. John, Pujol, Hinnebusch…).
“Pathogenic Yersinia species, which comprise the entero-pathogens Y. pseudotuberculosis and Y. enterocolitica as well as the plague agent Yersinia pestis, proliferate extracellularly in lymphoid tissues of animal hosts (Balada-Llasat & Mecsas, 2006; Viboud & Bliska, 2005). Therefore, the bacteria suppress phagocytosis, migration and immune signaling in resident cells of the innate immune system (Viboud & Bliska, 2005; Marketon et al., 2005).”*

We thank the reviewer for this valuable und insightful comment. We have addressed these points and amended the Introduction lines 72-75 to describe that *Yersinia* also has an intracellular niche.

***4)*** *This statement should be rephrased. As written implies it is only from the T3SS, there are also LPS, adhesions, YAPs, etc.. that the host cell may also interact with/sense during infection.
“Yersinia induced DAMPs are e.g. produced in response to membrane damage caused by the T3SS translocation pore or by deactivation of Rho GTPases through YopE and YopT (Schubert et al., 2020).”*

We thank the reviewer for this valuable comment. We have added LPS, heptose metabolites, T3SS pore and adhesins as PAMPs which are all known to trigger inflammatory signaling during *Yersinia* infection and describe this in Introduction lines 83-88. We also changed the description of responses triggered by the activity of T3SS effectors as effector-trigered immunity (ETI; lines 82-101) which refers to the host response upon disruption of cellular processes by bacterial effectors (Lopes Fischer et al., 2020 Nat Microbiol).

***5)*** *“Previous systematic studies of Yersinia effects on gene expression were conducted with gene arrays and cultured mouse macrophages infected for up to 2.5 h (Sauvonnet et al., 2002; Hoffmann et al., 2004).”
Again, the authors should include the studies of other Yersinia spp – one of which was an RNAi genome-wide screen in mouse macrophages to identify exploited pathways required for disease. Suggestion is to mention this with appropriate citations when applicable (Meagher, Sjostedt, Connor, Jett, Dersch, Bliska…).*

In response to the reviewers’ valuable comments, we have changed the Introduction lines 126 to 137 and cited other studies which performed gene expression analysis with all three pathogenic *Yersinia* species in various cell types and organisms. We did not include the study employing genome-wide RNAi screen during *Y. pestis* infection (Connor et al., 2018 mBio) because we particularly focused on gene expression and epigenetic changes which are modulated by *Yersinia* and therefore, we only cited studies present with gene expression analysis.

**Organization of results:**
***6)*** *As it is presented the authors focus mainly on the epigenetic changes without extracting the biological significance, which is present and interesting. A reorganization of the results and better contextualization of them would help immensely. The suggestion would be to organize the manuscript around the biological findings (already present in the text) cited below:
- WA314 suppresses PAMP-LPS-induced histone modifications.
All the data that illustrates this point should be grouped in one section (including promoters and enhancers), and functional analysis should be added. Indeed, in the context of the already known signaling suppression by Yersinia, it would be interesting to see the changes at the chromatin level, implying more than just suppression of gene activation.

- YopP but not YopM contributes to epigenetic changes produced by WA314.
Again, the most of the data is there, the authors need to spend more time on the functional analysis of the genomic regions differentially regulated by these two factors.

- Epigenetic modifications do not correlate well with transcriptional changes.
This message is not a problem but more an interesting finding. Indeed, this shows that beyond gene expression, there are “silent” chromatin modifications that could have a large impact on other physiological outcomes than infection. In line with this, the message that chromatin modifications correlate with expression should be removed, along with figure 4J. In this section the genes could be sub-classified to give biological meaning and importance to the genes in each category.
o Upregulated genes in WAC or WAC13
o Upregulated genes that are associated with HM at promoters
o Upregulated genes that are associated with HM at enhancers
o Upregulated genes that are associated with HM at promoters and enhancers*

*- Reprogramming of the Rho GTPase pathway.
Could the analysis be taken one step further to determine if a common transcription factor could be regulating all the genes in this category?*

We thank the reviewer for these useful suggestions, many of which will make the paper more easy to read and understand. We have made numerous changes in the manuscript accordingly:

i) We have grouped all figures and text about histone modification changes at promoters and enhancers in one figure (Fig 2), describing that the main activity of *Yersinia* effectors is suppression of PAMP chromatin induced changes.

ii) For the analysis of YopM and YopP effect we have added more functional analysis showing YopP regulation of gene expression and histone modifications through MAPK inhibition with qPCR experiments for ABI1 gene (S6E, G Figs). Moreover, we also added immunofluorescence experiments showing that YopP dependent redistribution of ABI1 and actin is reversed by the treatment with p300 histone acetyltransferase inhibitor, which reduces H3K27ac (Fig 5J, S6G Fig). However, we are planning to perform a much more in depth analysis including functional analysis of YopM and YopP activities in a future study and therefore feel that this issue is beyond the scope of this study.

iii) We have grouped together figures describing gene expression changes obtained with RNA-seq and the analysis of association between gene expression and histone modifications in the new Fig 3. As proposed, we show genes that associate with promoters **or** enhancers in Figs. 3D, G, and S3 Fig and genes that associate with **both**, promoters **and** enhancers in Fig 3F. We describe and show that many histone mark changes do not correlate with gene expression, which we also consider highly interesting. However, the main focus of our study was to investigate chromatin reorganization/ChIP-seq changes that correlate with gene expression/ RNA-seq changes because we were searching for new regulatory aspects of gene regulation during *Yersinia* infection. Therefore, all further analyses in this section were done for genes whose expression correlated with promoter and/or enhancer changes. As proposed, we did remove the scheme showing that histone modifications and gene expression correlate (Fig 4J in the previous version).

iv) We also find the concept that *Yersinia* induces a lot of “silent” histone modifications very interesting, which is briefly discussed in the Discussion lines 572-576 and should be investigated further in the future.

v) To emphasize the main biological finding of this study that histone modifications at promoters and enhancers represent another major level of gene regulation during *Yersinia* infection we have added a summary schematic in Fig 6.

vi) As also proposed by the reviewer, we had performed an analysis of potential transcription factor motif enrichment analysis for Rho GTPase pathway but we were not able to detect any strongly enriched motifs for a common regulator.

***7)*** *In general, there is a substantial amount of Yersinia work dedicated to signaling (MAPK, PI3K/AKT, Caspase(s) etc…) and trafficking (Rabs, VAMPs, SNAREs etc..) pathways exploited by this bacteria for pathogenesis that is not discussed in terms of this chromatin work. Include this contextual knowledge would strengthen the manuscript across all domains of Yersinia pathogenesis research, and in the context of what/how bacterial induced chromatin remodeling may interplay with these findings.*

We agree with this valuable comment and think that *Yersinia* might well target other pathways through histone modifications and gene expression. We therefore have included a short analysis of gene expression and chromatin modification changes for the mentioned pathways, which are also targeted by *Yersinia* such as MAPK and trafficking in the Results lines 329-338, S4C, D Figs and Table S16. A more detailed in-depth analysis of these and other pathways could be performed in the future. All the RNA-seq and ChIP-seq data will be publicly available, therefore further analysis of specific pathways can be also performed by anyone interested.

***8)*** *The terminology of the “suppression”, “prevention” profiles are very confusing and should be replaced or removed. Similarly, the scheme in figure 2B should be removed as it is confusing.*

We appreciate your opinion but we believe that this terminology is very helpful for reading and comprehending the complex matter of this study. Please refer also to the comment of Reviewer 2 in Part I concerning the profiles. However, following your suggestion we have removed the scheme in Figure 2.

**Data interpretation:** *There are many general conclusions that are drawn from the results that are overinterpreted.*

***9)*** *The title should be revised. For example, “Chromatin reorganization in macrophages upon Y. enterocolitica infection” or a more specific biological question. “Epigenetic reprogramming” is too strong of a statement here.*

We changed the title following this comment to “*Yersinia* remodels epigenetic histone modifications in human macrophages”.

***10)*** *In the abstract and author summary: “Y. enterocolitica profoundly reorganizes HM to reprogram key gene expression of the innate immune response”. This statement makes it sound as if Yersinia actively manipulates the host for HM. However, the authors describe in the manuscript how a big part of the response is common to LPS exposure. Also, chromatin reorganization could be a response of macrophages to Y. enterocolitica, not at active reprograming of the bacteria to the host. This statement is mentioned again at the end of section 1 and in the discussion.*

Following this comment we have made changes across the manuscript toning down the parts where we stated that bacteria actively reprogram host chromatin. Yet, we want to emphasize that many chromatin changes induced by LPS/PAMPs are actively reorganized by the virulence plasmid-encoded factors of the bacteria and a small fraction of changes is actively regulated by the virulence factors without any prior effects of LPS/PAMPs (Figs 2, 3A).

***11)*** *In the introduction: “Histone modifications determine DNA accessibility and transcriptional programs”. Determine is a very strong word. Influence is a better word, as the correlation between HM and transcription is not black or white. Also, in the introduction H3K4me3 is referred as a promoter mark but this mark also gets enriched in gene bodies of active genes.*

We agree that the correlation between histone marks and DNA accessibility and transcription is not always clear and have corrected “determine” to “influence” in the Introduction line 113. H3K4me3 is primarily known as a hallmark of active genes at transcriptional start sites, therefore we just mention this main feature of H3K4me3 in the Introduction (line 115). Clearly, H3K4me3 can also be found enriched in gene bodies or at enhancers at low levels (Chen et al., 2015 Nat Genet).

**Reviewer #2:**

***1.*** *Although the overall sets of data are largely descriptive, they give important information and reveal a new level of complexity of Yersinia-triggered macrophage responses which are of great interest for the community. However, from what I understood from their rather cryptic description about the quantitative set-up (biological and technical replicates) of the experiment and analyses, the entire set of analyses is based on two biological replicates which makes a statistical analysis and statements about the significance of identified histone modification and gene expression changes rather difficult.
In the material method section, they state that for RNA-seq analysis two representative replicates are shown for each sample (p. 32 last line), but it is unclear how many biological and technical replicates were included into the entire analysis, and which were included into the analysis of differential expression with DESeq2. A more detailed description of the biological and technical replicates used for the histone modification and gene expression analyses (statistical analysis - p values) is required to allow a proper judgment for the significance of the data. This seems particularly important as histone modification data and expression data between the documented two representative data sets seem to vary considerably with respect to several illustrated responses (Fig. 4A for WAC and WA314; Fig. 5D: WAC positive regulation, WA314 Wnt signalling, Fig, 5E WA314; Fig. 6B and 6E for WA314, Fig, S4 E WAC and WA314 etc.).*

We thank the reviewer for this comment and apologize that we were unconcise concerning the technical and biological replicates employed in our study. Throughout the study we used 2-4 biological replicates (macrophages from different donors, not pooled) for the experiments and in all cases they showed excellent reproducibility (see explanations and figures below). All available replicates were included in the analysis for the detection of DEGs from RNA-seq and differentially enriched regions from ChIP-seq. In any case, only genes and regions which were statistically significant (≥ 2-fold change, adjusted P-value ≤ 0.05) were used for the analysis as described in the Materials and Methods for RNA-seq DEG analysis and ChIP-seq differential enrichment analysis. We have now added more information about the replicates employed in Materials and Methods for RNA-seq and ChIP-seq and a supplementary Table S17 describing all the replicates used for the analysis for clarification. It is good standard that a minimum of 2 or more replicates are used for ChIP-seq and RNA-seq studies, e.g., analyzing inflammatory macrophage responses, as exemplified in the studies below (Kang et al., 2019; Park et al., 2017; Novakovic et al., 206; Xue et al., 2014; Bambouskova et al., 2018; Wang et al., 2019). Furthermore, ENCODE and other guidelines recommend a minimum of 2 replicates for RNA-seq and ChIP-seq studies (Kidder et al., 2011; Landt et al., 2012; Liu et al., 2013; Davis et al., 2018). These studies show that higher number of replicates in RNA-seq is needed to detect increased number of differentially expressed genes and subtle gene expression changes, but at least 2 replicates are sufficient to detect most highly differentially expressed genes (Liu et al., 2014; Schurch et al., 2016; Lamarre et al., 2018). Moreover, DESeq2 tool, which was employed in our study, is particularly suitable for analyzing RNA-seq data with low number of replicates (≤3) showing robust false positive and false negative rates (Schurch et al., 2016; Lamarre et al., 2018). Also, for ChIP-seq analysis diffReps tool used in our study shows reliable results at detecting sites of differential enrichment for histone marks with 2 replicates (Shen et al., 2013; Steinhauser et al., 2016). Therefore, we are absolutely positive from our own analyses and the literature that we have analyzed a sufficient number of replicates in our study.

Kang K, Bachu M, Park SH, et al. IFN-γ selectively suppresses a subset of TLR4-activated genes and enhancers to potentiate macrophage activation. *Nat Commun*. 2019;10(1):3320. Published 2019 Jul 25. doi:10.1038/s41467-019-11147-3

Park SH, Kang K, Giannopoulou E, et al. Type I interferons and the cytokine TNF cooperatively reprogram the macrophage epigenome to promote inflammatory activation. *Nat Immunol*. 2017;18(10):1104-1116. doi:10.1038/ni.3818

Novakovic B, Habibi E, Wang SY, et al. β-Glucan Reverses the Epigenetic State of LPS-Induced Immunological Tolerance. *Cell*. 2016;167(5):1354-1368.e14. doi:10.1016/j.cell.2016.09.034

Xue J, Schmidt SV, Sander J, et al. Transcriptome-based network analysis reveals a spectrum model of human macrophage activation. *Immunity*. 2014;40(2):274-288. doi:10.1016/j.immuni.2014.01.006

Bambouskova M, Gorvel L, Lampropoulou V, et al. Electrophilic properties of itaconate and derivatives regulate the IκBζ-ATF3 inflammatory axis. *Nature*. 2018;556(7702):501-504. doi:10.1038/s41586-018-0052-z

Wang C, Nanni L, Novakovic B, et al. Extensive epigenomic integration of the glucocorticoid response in primary human monocytes and in vitro derived macrophages. *Sci Rep*. 2019;9(1):2772. Published 2019 Feb 26. doi:10.1038/s41598-019-39395-9

Shen L, Shao NY, Liu X, Maze I, Feng J, Nestler EJ. diffReps: detecting differential chromatin modification sites from ChIP-seq data with biological replicates. PLoS One. 2013;8(6):e65598. Published 2013 Jun 10. doi:10.1371/journal.pone.0065598

Kidder BL, Hu G, Zhao K. ChIP-Seq: technical considerations for obtaining high-quality data. Nat Immunol. 2011;12(10):918-922. Published 2011 Sep 20. doi:10.1038/ni.2117

Landt SG, Marinov GK, Kundaje A, et al. ChIP-seq guidelines and practices of the ENCODE and modENCODE consortia. Genome Res. 2012;22(9):1813-1831. doi:10.1101/gr.136184.111

Liu Y, Zhou J, White KP. RNA-seq differential expression studies: more sequence or more replication?. Bioinformatics. 2014;30(3):301-304. doi:10.1093/bioinformatics/btt688

Schurch NJ, Schofield P, Gierliński M, et al. How many biological replicates are needed in an RNA-seq experiment and which differential expression tool should you use? [published correction appears in RNA. 2016 Oct;22(10 ):1641]. RNA. 2016;22(6):839-851. doi:10.1261/rna.053959.115

Davis CA, Hitz BC, Sloan CA, et al. The Encyclopedia of DNA elements (ENCODE): data portal update. Nucleic Acids Res. 2018;46(D1):D794-D801. doi:10.1093/nar/gkx1081

Lamarre S, Frasse P, Zouine M, et al. Optimization of an RNA-Seq Differential Gene Expression Analysis Depending on Biological Replicate Number and Library Size. Front Plant Sci. 2018;9:108. Published 2018 Feb 14. doi:10.3389/fpls.2018.00108

Steinhauser S, Kurzawa N, Eils R, Herrmann C. A comprehensive comparison of tools for differential ChIP-seq analysis. Brief Bioinform. 2016;17(6):953-966. doi:10.1093/bib/bbv110

We are aware of the variation within the data sets in the visualized examples of RNA-seq gene counts. There are some factors which contribute to this variation. Our biological macrophage replicates come from different human donors, which could be a reason for the observed heterogeneity. Very importantly however, this heterogeneity mostly affected the effect size (how strong the change is) and not whether the effect occurs or not. Furthermore, in our clustering analysis we used all differentially expressed genes between mock, WAC and WA314 (comparisons WAC vs mock, WA314 vs mock, WAC vs WA314), which means that not all genes presented in the heatmaps are significantly different between every comparison. E.g., a presented gene might be a DEG between WAC vs mock but not between WAC vs WA314. A fraction of the presented genes are not DEGs between WAC vs WA314 due to the inconsistencies between replicates or not full suppression of the WAC-induced changes by WA314, which will appear as variation in the heatmaps. Further, wild type strain WA314 does not completely suppress WAC (LPS)-induced changes and the expression levels of WA314 are mostly between mock and WAC. Finally, how strongly WA314 suppresses WAC often depends on the biological replicate and this presents another source of variation.

PCA plots of all H3K27ac **(left)** and H3K4me3 **(right)** ChIP-seq sample replicates used for the analysis. Different colors and shapes indicate different samples and replicates, respectively. WAP (WA314), dYopM (WA314△YopM), dYopP (WA314△YopP).

**Right:** Figure showing heatmap of RNA-seq sample clustering based on Euclidean distance where dark blue indicates smaller sample distances and larger similarity.

***2.*** *In the figures, exact numbers of genes, promoters and enhancers which are modified with respect to histones and/or gene expression upon the infection with applied Yersinia strains are given. However, (in line with the statistical analysis) the validity of the data is unclear. Have the authors performed assays to validate the detected histone modification marks and the gene expression data, which is important for the reliability of the data, e.g. proof of principle experiments for the Rho proteins?*

We thank the reviewer for this comment. In our analysis we only used statistically significantly (≥ 2-fold change, adjusted P-value ≤ 0.05 from DESeq2 and diffReps analysis) changed genes and regions. We performed a validation of histone marks from ChIP-seq data and gene expression from RNA-seq data which is now included in S2 Fig, S6E, F Figs and Fig 5F.

**Reviewer #3:**

*Page 24, the ABI1 protein western blot data (Fig. S5E) should be move to a main figure and some quantification should be provided to confirm the differences are reproducible. At a minimum the authors should show the results of the two experiments and show some semi-quantification.*

We thank the reviewer for this suggestion. We have moved the western blot data to Fig 5G, with quantification from 3 independent biological replicates.

*End of Page 24, what is the evidence "that the histone modification-driven reprogramming of ABI1 gene expression in fact can alter actin cytoskeleton organization in Yersinia infected macrophages"? The authors need to change this conclusion or provide evidence that histone modification is responsible for differential ABI1 gene expression.*

We thank the reviewer for this question. We have toned down our statement that reorganization of actin cytoskeleton is histone modification-driven. We have also added additional ChIP-qPCR and RT-qPCR analysis of ABI1 gene overall showing that changes in histone modifications, gene expression, protein levels and redistribution of actin cytoskeleton and ABI1 are associated with MAPK pathway activation, which supports the idea of histone modifications affecting ABI1 and actin cytoskeleton (Figs 5H, I, S6E, F Fig). We additionally show that redistribution of ABI1 and actin is reversed by treatment with p300 histone acetyltransferase inhibitor (Fig 5J) which reduces H3K27ac (S6G Fig). This experiment provides a more direct, albeit still not very specific connection of the regulation of actin through histone modifications. However, to clearly validate this idea other experiments that are far beyond the scope of this work such as directly targeting histone modifications at specific regions would be needed.

**Part III – Minor Issues: Editorial and Data Presentation Modifications**

**Reviewer #1:**

***1)*** *Please number the lines as this makes reviewing easier.*

We have numbered the lines.

***2)*** *The figure legends need to be more informative. For example it is hard to know what data was used to perform clustering figures (DEGs, genes in a specific caterogy, nearest gene to histone mark, etc.)*

We have described figure legends in more detail and think that they are clearer to understand now.

***3)*** *There are quite a few grammatical errors throughout which should be revised.*

We have tried to correct grammatical errors as far as possible.

***4)*** *HM referring to histone mark is problematic as it can also be human macrophage – please find a new term.*

We have noted your comment and changed “HM” to “HMod”.

***5)*** *A citation that macrophages produce TJs? This reviewer is unaware that mature macrophages made TJs.*

The observed junctions from our work, not necessarily tight junctions, have not been previously described in macrophages based on a literature search and therefore there is no citation added.

***6)*** *The quality of the images are very bad and sometimes impossible to read.*

Low resolution images were grouped at the end of the manuscript by the submission system and high resolution images are provided in the download link for each figure.

**Reviewer #2: Specific comments:**

***1.*** *p. 7, introduction, 2nd paragraph: the authors state that previous systemic analyses on gene expression were performed up to 2.5 h. In the present study the authors decided to analyze histone modification and gene expression changes after 6 h. Why? Do histone modifications need this long? The authors also state that most histone modifications are transient (p.7, last sentence of the 2nd paragraph). Are the observed histone modifications maximal at 6 h? What is known about the dynamics of this process?*

i) We have now included additional studies in the Introduction analyzing gene expression during *Yersinia* infection in general (lines 126-137), where in one of those studies mouse macrophages had been infected also for up to 4 h. Shorter infection times (2-4 h) have to be used for mouse macrophages because of induction of cell death at later time points.

ii) We chose 6 h of infection for gene expression analysis because at this time point we saw in a previous study strong modulation of gene expression in macrophages by *Yersinia* T3SS effectors (Berneking et al., 2016 PLOS Pathog) which included both primary and secondary response genes in TLR signaling. We had done also RNA-seq analysis after early 1.5 h time point which showed that both virulent and avirulent *Yersinia* induce similar gene expression changes.

iii) Time course ChIP-qPCR analysis of H3K4me3 and H3K27ac after 1 h, 3 h and 6 h of *Y. enterocolitica* WAC infection showed that these marks show the most changes at 6 h. This information is now included in S1C Fig.

*Moreover, are the histone modifications immediately visible in the gene expression pattern or are they somewhat delayed? If latter, could this also be one reason why observed correlation between histone modification and gene expression patterns is rather low (max. 30% for the Suppression profile). What about ChIP-seq – gene expression data from other studies - are the correlations in a similar range? These points should also be addressed/discussed.*

Chromatin changes in macrophages do not always associate with gene expression, however, they still appear important for macrophage immune response as they can prime cells to respond differently upon repeated stimulation (Park et al., 2017 Nat Immunol; Qiao et al., 2013 Immunity; Kang et al., 2019 Nat Commun). Moreover, gene transcription is defined by the sum of all gene-associated chromatin modifications and bound proteins, thus a change in one histone mark might not reflect the overall chromatin landscape and the transcriptional state. We have addressed this point in the Discussion lines 571-576. Furthermore, in general we saw that promoter H3K4me3 and H3K27ac showed much stronger association with gene expression than H3K27ac enhancer changes (S3 Fig). The reason for this could be that histone marks overlapping promoters are often co-transcriptionally deposited and can be easily assigned to the genes they regulate. However, enhancers often regulate genes from a very long distance and enhancers do not always regulate the closest genes in the vicinity. We assigned enhancers to the closest genes, which is a very common practice, if no data about genomic contacts is available, but it might not always be accurate.

We had done some additional ChIP-seq experiments for testing with H3K4me3 also at 1.5 h so we could compare histone mark and gene expression dynamics at 1.5 h and 6 h. From these experiments we saw that genes with increased expression at 1.5 h also show increased H3K4me3, the same pattern was observed for 6 h. For precise characterization of histone mark and gene expression dynamics more time points would be needed, but from our data so far we can conclude that in general histone marks appear before or during the induction of gene expression. This in line also with publications showing that H3K4me3 recruits transcriptional machinery (Lauberth et al., 2013 Cell; Vermeulen et al., 2007 Cell) or is deposited co-transcriptionally (Kuang et al., 2014 Nat Struct Mol Biol).

***2.*** *p. 8, results: 1st paragraph/p. 30-34, material and methods, Fig. 1 legends: in neither of these sections, the quantitative set-up of the experiments which are basis for their overall analysis is explained. How many biological and technical replicates were used to obtain the data. Were independent samples pooled? On p. 32 last sentence states…2 representative replicates are shown for each sample. What about the other replicates? Have they included into the data sets or are the given data sets from the two selected representative samples. Why were only these two selected?*

We have used 2-4 biological replicates (macrophages from different donors) for each sample without pooling the donors. As replicates showed good reproducibility, all of the collected replicates were included in the analysis for the detection of DEGs from RNA-seq and differentially enriched regions from ChIP-seq. The number of replicates differs between samples because some experiments were performed in different batches with different sample composition. For samples with 4 replicates only 2 replicates in the figures are shown for visualization purposes so that the number of replicates shown is the same for each sample. Supplementary tables contain counts for ChIP-seq and RNA-seq from all replicates. We have now added more information about the replicates in Materials and Methods for RNA-seq and ChIP-seq and a supplementary Table S17 describing all the replicates used for the analysis for clarification.

***3.*** *p. 8, l. 15, results: …H3K4me1 …. Is also found with lower levels are promoters…similar to H3K4me3, it should be added whether this indicates active or inactive promoters.*

We have removed description of H3K4me1 as a promoter mark because H3K4me1 is a hallmark of enhancers and is discussed in studies analyzing histone modifications in macrophages and in this study in the context of enhancers.

Depending on the context H3K4me1 at promoters can indicate repressed, active or poised state (Bae & Lesch et al., 2020 Front Cell Dev Biol; Cheng et al., 2014 Mol Cell).

***4.*** *p. 10, l. 2-3, results: Sentence starting … ‘Overall, WAC and WA314…’ seems incomplete.*

We have removed the sentence and the corresponding figure (S1C Fig) when revising the manuscript.

***5.*** *p. 9-12, results: This part is very descriptive and mostly lists the histone modifications (percentage) observed for the genes/promoters/enhancers in the different strains without an explanation of the consequences and could be shortened.*

We have shortened the description for Fig 1E following your comment.

***6.*** *p. 13, results: 1st paragraph/p. 30-34, material and methods, Fig. 4 legends: in neither of these sections, the quantitative set-up of the experiments which are basis for their overall analysis of the gene expression patterns is explained. How many biological and technical replicates were used to obtain the data. Were independent samples pooled?*

Samples were not pooled and each replicate represents an individual macrophage donor. We have added Table S17 to supplementary data listing all the biological replicates used for the analysis.

***7.*** *Table S2, S3, S5, S6, S9 please describe rows (e.g. DoA, DoB, what parameters are represented by the numbers, RPKM for RNA-seq?) and add statistical analysis (p-value etc.). The ordering by chromosome location is okay, but additional sheets in which the genes are ordered with respect to the log2fold change would be helpful.*

We have explained the columns of tables in more detail and added description of parameters represented by the numbers and statistical analysis, where it was missing. We believe that all the available data in the tables can be reorganized and sorted easily in a way which is the most relevant for the readers and therefore we did not change the order of columns or genes.

***8.*** *p. 16, l. 14, 15: please explain abbreviation TF.*

We have explained the abbreviation in line 339.

***9.*** *p. 17, l. 10: I suggest to exchange ‘there’ against ‘this’*

We believe that “this” fits better in this context.

***10.*** *p. 25, paragraph 4: the bacteria’s T3SS effectors block PAMP-associated deposition or removal – T3SS effectors should be replaced by virulence-plasmid-encoded pathogenicity factors. It is possible that other non-effector virulence factors such as YadA, which also activate MAPK pathways etc., are also implicated in reprogramming.*

We have addressed this comment and changed “T3SS effectors” to “virulence plasmid-encoded factors” in line 256.

***11.*** *p. 26 3rd paragraph…We could exclude YopM as one of these effectors, because it did not alter any of the histone modifications – is this really true? See p. 21 3rd sentence from the buttom, in which the author state, that 31 histone modifications were differentially regulated between the yopM mutant and the wildtype.*

YopM regulates histone modifications at an extremely small number of regions especially when compared to YopP (29 vs 5778 regions). So, we considered regulation of H3K4me3 and H3K27ac by YopM negligible. We rephrased sentence to “We could exclude YopM as one of these effectors, because it did not alter close to any of the histone modifications” in line 592.

**12.** *p. 27-28, last and first sentence: the references added to this sentence do not document, that expression of the mentioned Rac1 associated proteins were suppressed by the bacteria…. they document that they can take over Rac1 functions, please clarify.*

We have made the suggested correction (lines 621-624).

**13.** *p. 30, the authors used an MOI of 100 to infect the macrophages. This is okay to obtain information about how Yersinia can manipulate human macrophages. Just for curiosity? Which MOI is expected for an in vivo infection (i.e. infections of mice, humans)? If a lower MOI is more likely, do you expect the histone modification and gene expression changes to be identical, just less pronounced, or is it likely to have a different set of histone-modified.*

It is challenging or near to impossible to predict the expected MOI during an in vivo infection, also because close by cells could be associated with very different numbers of bacteria. Our feeling is that an in vivo MOI in general would be less than 100. We think that during infection with lower MOI the same histone modifications would be observed, however, fewer host cells might be affected as seen in the analysis of cells positive for translocated effectors with different MOIs in S1B Fig. Furthermore, the effect might be less pronounced or take longer to get established as it has been observed for *Yersinia* induction of cell death and cytokine secretion with different MOIs (Sarhan et al., 2018 Proc Natl Acad Sci USA; Muendheim et al., 2021 Nat Commun).

**Reviewer #3:**

*The manuscript did not have line numbers to help with reviewing.*
We have added the line numbers now.

*Page 2, change "immune cells" to "macrophages"*

We have changed "immune cells" to "macrophages".

*Page 5, "Further, YopP/J, and YopM inhibit the inflammatory responses triggered by the PAMPs or the damage associated molecular patterns (DAMPs) elicited by the bacteria." This sentence and related text need to be revised, as the authors are equating effector-triggered immune responses, which YopM does inhibit, with DAMP-triggered immune responses.*

We have addressed this point and made corrections (lines 80-101).

*Page 6, define "H3K27ac"*

We have now defined it in the line 115.

*Page 19, the terminology "actually active" is awkward.*

We have removed “actually” in the line 408.
